# Coronary Artery Calcification Is Related to Inflammation in Rheumatoid Arthritis: A Long-Term Follow-Up Study

**DOI:** 10.1155/2016/1261582

**Published:** 2016-08-28

**Authors:** Bengt Wahlin, Thomas Meedt, Fredrik Jonsson, Michael Y. Henein, Solveig Wållberg-Jonsson

**Affiliations:** ^1^Department of Public Health and Clinical Medicine/Rheumatology, Umeå University, 901 87 Umeå, Sweden; ^2^Department of Public Health and Clinical Medicine, Umeå University, 901 87 Umeå, Sweden; ^3^Department of Public Health and Clinical Medicine/Cardiology, Umeå University, 901 87 Umeå, Sweden

## Abstract

*Objective*. A long-term follow-up of patients with rheumatoid arthritis (RA) to evaluate factors related to coronary artery calcification (CAC).* Methods*. All 22 eligible patients (4 males/18 females, mean age 65 years, and RA-duration 30–36 years) from the original (baseline; *n* = 39) study of atherosclerosis were included. Inflammation, cardiovascular risk factors, and biomarkers were measured at baseline. At follow-up 13 years later, CAC was assessed by computed tomography (CT) and the grade of inflammation was measured. Multivariate analysis of differences between patients with low (0–10) and high CAC (>10) was done by orthogonal projection to latent structures (OPLS).* Results*. Ten patients had CAC 0–10 and 12 had >10 (range 18–1700). Patients with high CAC had significantly higher ESR (24.3 versus 9.9 mm/h) and swollen joint count (2 versus 0). The OPLS models discriminated between patients having high or low CAC. With only baseline variables, the sensitivity was 73% and the specificity 82%. The model that also included inflammatory variables from follow-up had a sensitivity of 89% and a specificity of 85%. Exclusion of baseline intima media thickness and plaque from the latter model modestly reduced the accuracy (sensitivity 80% and specificity 83%).* Conclusions*. CAC is related to inflammation in patients with RA.

## 1. Introduction

Morbidity and mortality due to cardiovascular disease (CVD) are increased in rheumatoid arthritis (RA) [1–3] reflecting the additional burden of atherosclerosis [4–6]. In subclinical stages, atherosclerosis can be assessed by carotid ultrasound, where plaques are detected and the intima media thickness (IMT) is measured. Increased IMT is an early sign of atherosclerosis and has been found to predict future CVD in the general population [7] as well as in RA [8, 9]. Another way to assess subclinical atherosclerosis is computed tomography (CT) of the coronary arteries, where the amount of calcium is quantified using the Agatston method [10]. Coronary artery calcium (CAC) has been considered a risk marker for and shown to predict future CVD in both the general population [11, 12] and patients with inflammatory diseases including RA [13]. As is the case for other measures of atherosclerosis, the prevalence of CAC is higher in RA than in the general population [14–18]. These earlier studies are cross-sectional, with conflicting results regarding the relation between measures of inflammation and CAC. Also for other measures of atherosclerosis, the association with inflammation is somewhat uncertain [19].

In a previous study, our group demonstrated a higher prevalence of atherosclerosis in patients with seropositive RA compared to matched controls [6]. In that study, extensive laboratory investigations were made, including biomarkers of endothelial activation, hemostasis, inflammation, and blood lipids. In view of few long-term studies on the progression of atherosclerosis in patients with RA, a follow-up of the patients was undertaken 13 years after the first investigation to evaluate factors important for development of atherosclerosis, as measured by CAC. Thus, the aims of this follow-up study were to evaluate (i) the prevalence of CAC, (ii) the association between baseline ultrasound findings and biomarkers, respectively, and future CAC, and (iii) the association between inflammation and CAC.

## 2. Materials and Method

### 2.1. Baseline

Thirty-nine patients from northern Sweden with seropositive RA who fulfilled the 1987 American College of Rheumatology classification criteria [20] were examined in 1997-1998 for the presence of atherosclerosis and markers of atherogenesis and thrombogenesis [6, 21, 22]. The group comprised all eligible patients, who were reported with new onset of seropositive RA at the Department of Rheumatology in Umeå between 1974 and 1978 and at the time of examination were less than 66 years old. The patients had been treated according to clinical praxis. At the time for the baseline examination, the disease duration was 19–23 years. Using B-mode ultrasound, carotid IMT was measured and the extent of plaques in the common carotid and femoral arteries was detected. In addition, blood samples were collected and analysed. Markers of inflammation included erythrocyte sedimentation rate (ESR), C-reactive peptide (CRP), soluble receptor of interleukin-2 (IL2sR), interleukin-6 (IL-6), and haptoglobin. Blood lipid analyses included cholesterol, high-density lipoproteins (HDL), low-density lipoproteins (LDL), triglycerides (TG), lipoprotein(a) (Lp(a)), and calculation of LDL/HDL ratio. The presence of antibodies with affinity for oxidized LDL of classes IgA (ox-LDL IgA), IgG (ox-LDL IgG), and IgM (ox-LDL IgM) was assessed as well as antibodies directed against malondialdehyde-modified LDL of classes IgA (MDA-LDL IgA), IgG (MDA-LDL IgG), and IgM (MDA-LDL IgM). Endothelial activation and adhesion molecules were analysed, including soluble intercellular adhesion molecule-1 (ICAM-1), E-selectin, and circulating immune complexes (CIC). Hemostatic factors analysed were von Willebrand factor (vWF), plasminogen activator inhibitor-1 mass (PAI), tissue plasminogen activator antigen (tPA), D-dimer, fibrinogen, and anti-cardiolipin antibodies of classes IgA (aCL IgA), IgG (aCL IgG), and IgM (aCL IgM). Leptin and homocysteine levels were also analysed. Sampling and analytical procedures have been described before in detail [6, 21]. Medical records were studied to calculate a retrospective accumulated disease activity score according to Baecklund [23]. In the present study, the previous study is referred to as “baseline.”

### 2.2. Follow-Up

#### 2.2.1. Patients

All patients from the baseline study alive and living in Sweden were invited to participate in the current follow-up study. Since the baseline visit, the patients had been treated with antirheumatic agents according to clinical praxis, including various synthetic DMARDs, biologic treatment, NSAID, and corticosteroids. Seven patients had died and one had emigrated since baseline. Of the remaining patients, seven refused to participate, most due to severe disability. Of the remaining 24 patients, two were assessed clinically but not by coronary CT and are not included in the present study. The remaining 22 patients (18 females; 4 males) constitute the subjects of this follow-up study, which was performed after a median of 13.0 years (range 12.4–14.0) from baseline. The study was approved by the local board of ethics at Umeå University and informed consent was obtained from the patients.

#### 2.2.2. Clinical Examination and Disease Activity

In each patient, 28 joints were examined for the presence of swelling or tenderness. The patients also completed the health assessment questionnaire (HAQ) [24], visual analogue scales (VAS) for pain, and global assessment and their current medication was registered. The composite activity index DAS28 [25] was calculated from global VAS, tender joints, swollen joints, and ESR.

#### 2.2.3. Blood Analyses

At the follow-up visit, fasting blood samples were collected and analysed using standard laboratory methods at the laboratory of clinical chemistry, Umeå University Hospital. Markers of inflammation (ESR, CRP, and haptoglobin) were measured. CRP was analysed with low-sensitive method, with lower limit <5 mg/mL. Changes in ESR, CRP, and haptoglobin (delta values) were calculated by subtracting baseline value from the follow-up value.

#### 2.2.4. Arterial Imaging

**Figure 1:**
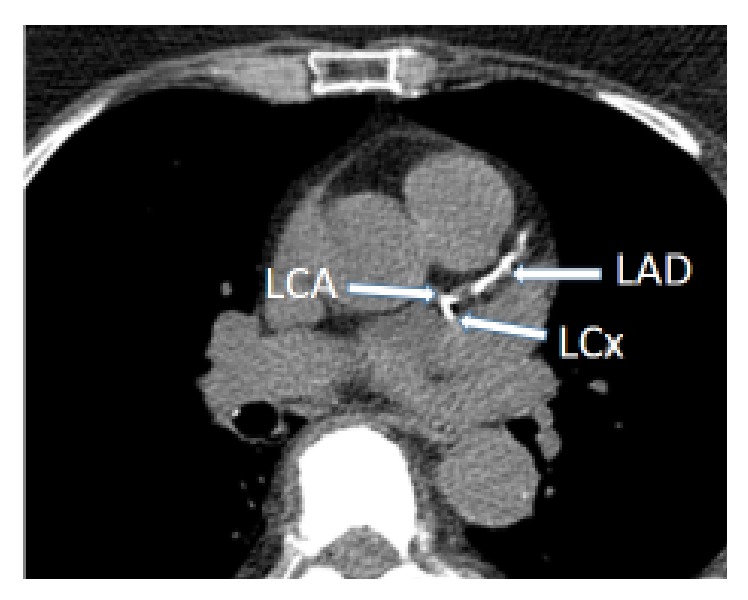
CT scan. Calcifications are seen in the bifurcation of the left coronary artery (LCA), the proximal part of left circumflex artery (LCx), and the left anterior descending artery (LAD). Noncalcified parts are visible both proximal and more distal in LAD, seen as low-attenuating areas between the calcified parts.

Assessment of CAC was performed by multidetector computed tomography (LightSpeed VCT, GE Healthcare, Milwaukee, WI, USA). The CT scan was ECG-gated at 65% of R-R-interval.  Figure 1 is a CT scan illustrating calcified lesions in one of the patients. The images were analysed on a workstation (Advantage 4.4, GE Medical systems, Milwaukee, WI, USA) and CAC was detected by a dedicated software (SmartScore version 4.0, GE Medical systems). The CAC score was quantified by the Agatston method [10]. By this method, the threshold for calcification is 130 Hounsfield units (HU) with a minimum of 2 connected pixels. The score for each calcified lesion is calculated by multiplying the area of calcification with a factor based on the peak attenuation of the lesion. The CAC score for a patient is the sum of scores from every lesion in the coronary arteries. Patients were classified as having either low CAC (CAC 0–10) or high CAC (score > 10). This classification was based on the study by Budoff et al. [11], where patients with CAC 1–10 had only slightly increased mortality compared to CAC = 0, but CAC > 10 was an independent strong predictor of mortality.

### 2.3. Statistical Analysis

Demographic, clinical, and laboratory data are presented as proportions or medians with interquartile ranges (Q1–Q3). Comparisons between groups were done using the Mann–Whitney* U* test with two-tailed exact method. *p* values <0.05 were considered significant. Multivariate discriminant analysis was performed using the orthogonal projection to latent structures discriminant analysis (OPLS) algorithm [26]. This method is designed to find quantitative relations between descriptive variables and response variables. The basis for OPLS is transition from a large number of descriptive variables to a small number of vectors representing latent variables. The first latent variable (vector 1) is the latent variable that best explains the variation in the response variable, in this case high or low CAC. The successive latent variables are orthogonal to vector 1, since they are independent of the response variable (in this study CAC status) but still represent information that varies in a nonrandom pattern, although it does not discriminate the two groups of patients from each other. When a new OPLS model was tested, variables without influence on the model were excluded (VIP < 0.5). This exclusion was repeated until every variable included in the model had VIP ≥ 0.5. Receiver operating characteristic (ROC) analysis was made for each model to evaluate its sensitivity and specificity. OPLS was performed using Simca version 13.0 (Umetrics, Umeå, Sweden). Other statistical analyses were performed using SPSS version 21 (IBM SPSS INC, Chicago, Illinois, USA).

## 3. Results

Demographic characteristics, cardiovascular risk factors, and inflammatory variables at follow-up are presented in Table 1.

### 3.1. Coronary Artery Calcification

CAC was assessed in 22 patients. Eight patients (36.4%) had no detectable CAC. Fourteen patients had scores between 6 and 1700 with a median value of 281 (33–490). Ten patients were classified into the low CAC group (0–10) and 12 into the high CAC group (>10).

### 3.2. Univariate Analysis

The levels of inflammatory variables at follow-up were higher in patients with high CAC compared to those with low CAC (Table 2). The differences were statistically significant for DAS28, ESR, and swollen joint counts.

### 3.3. Multivariate Analysis

**Figure 2:**
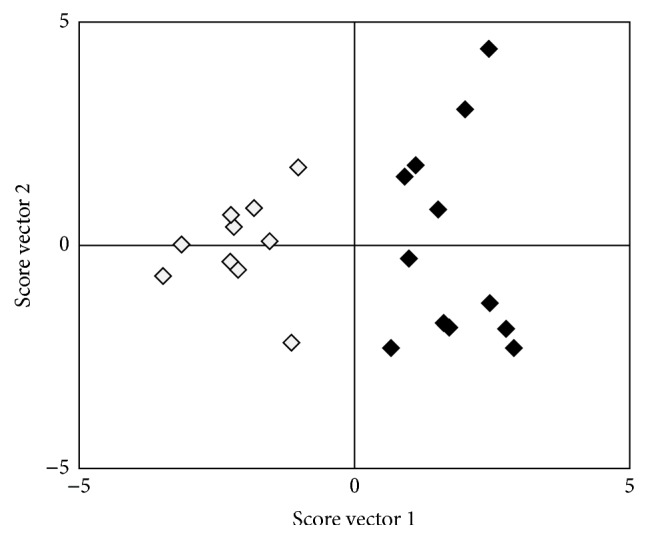
OPLS score scatter plot model 1. The black diamonds represent patients with CAC > 10 and the grey diamonds patients with CAC 0–10. Vector 1 (*x*-axis) is the latent variable that best separates patients with CAC 0–10 from patients with CAC > 10. The score for a patient is calculated from the values of the variables included in the model. See Materials and Method for further explanation. CAC: coronary artery calcification.

**Figure 3:**
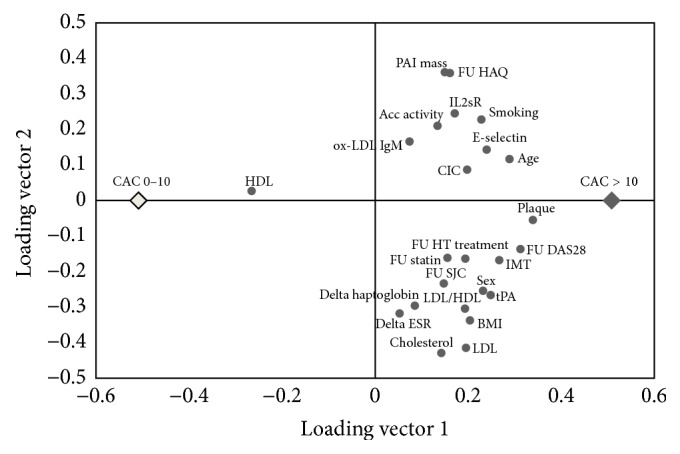
OPLS loading plot model 1. The dots represent the loadings of the single variables in each of the two vectors (latent variables) in the OPLS model. This plot displays how the variables are related to each other and to CAC status. The distance from origo is proportional to the importance in the OPLS model. Acc activity: accumulated disease activity. BMI: body mass index. CAC: coronary artery calcification. CIC: circulating immune complexes. DAS28: 28-joint disease activity score. ESR: erythrocyte sedimentation rate. FU: follow-up. HAQ: health assessment questionnaire. HDL: high-density lipoproteins. HT: hypertension. IL2sR: soluble receptor of interleukin-2. IMT: carotid intima media thickness. LDL: low-density lipoproteins. PAI: plasminogen activator inhibitor-1 mass. SJC: swollen joint count. VAS: visual analogue scale.

Three OPLS models with different settings of variables were tested. The first model tested (model 1) initially included all variables from baseline and follow-up measures of joint counts, HAQ, DAS28, antihypertensive treatment, and statin treatment together with change from baseline (delta values) of ESR, CRP, and haptoglobin.  Figure 2 is a score scatter plot showing the results from this OPLS model, visualizing how patients with high and low CAC are separated by the information in the variables included in the model. The loading plot (Figure 3) gives a survey of how the variables are related to each other and how they contribute to the discrimination between patients with high CAC and low CAC. The more to the right a variable is plotted, the stronger the association with high CAC is. HDL is the only variable plotted on the left half, since it is the only variable with a positive relation to low CAC. *R* ^2^ for this model was 0.87, meaning that 87% of the outcome is explained by the included variables. Subsequent ROC analysis displayed a sensitivity of 89% and a specificity of 85% in discriminating between high and low CAC. When the baseline ultrasound variables (IMT and plaque) were omitted from the model, *R* ^2^ was 0.86, sensitivity was 80%, and specificity was 83%.

**Figure 4:**
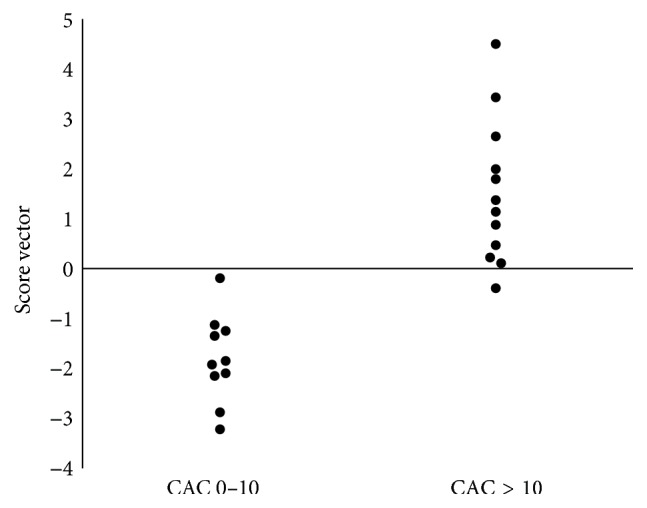
OPLS score vectors model 2. In the left column are the OPLS scores for patients with CAC 0–10 and in the right column the OPLS scores for patients with CAC > 10. In this model only one latent variable (vector) was identified. CAC: coronary artery calcification.

**Figure 5:**
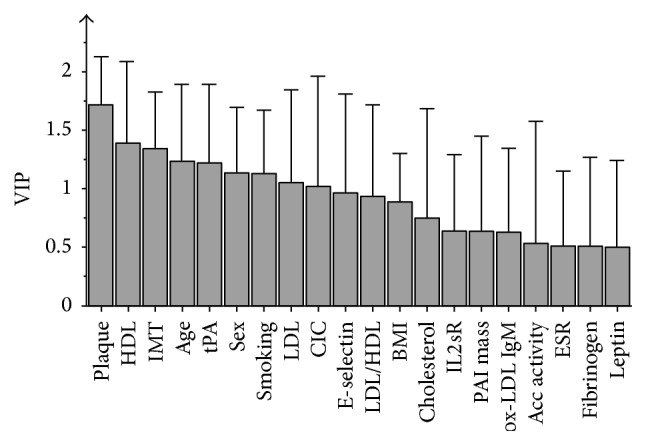
OPLS variable importance in projection (VIP) model 2. The numerical value for a variable represents an estimation of its importance in the model, that is, to what grade it discriminates between the two groups of patients. Whiskers illustrate the upper limit of bootstrapped confidence intervals. Acc activity: accumulated disease activity. BMI: body mass index. CIC: circulating immune complexes. ESR: erythrocyte sedimentation rate. FU: follow-up. HAQ: health assessment questionnaire. HDL: high-density lipoproteins. IL2sR: soluble receptor of interleukin-2. IMT: carotid intima media thickness. LDL: low-density lipoproteins. ox-LDL: oxidized LDL. PAI: plasminogen activator inhibitor-1 mass. tPA: tissue plasminogen activator.

In OPLS model 2 (Figures 4 and 5), initially all baseline variables were included, but not the follow-up variables reflecting current inflammation, nor delta values. This model was tested to investigate if the baseline variables could predict CAC status 13 years later.  Figure 4 visualizes how patients with high or low CAC are separated by the score vectors from this model. The VIP is presented in Figure 5. All variables except HDL and leptin were positively related to high CAC. *R* ^2^ for this model was 0.67, sensitivity 73%, and specificity 82%.

**Figure 6:**
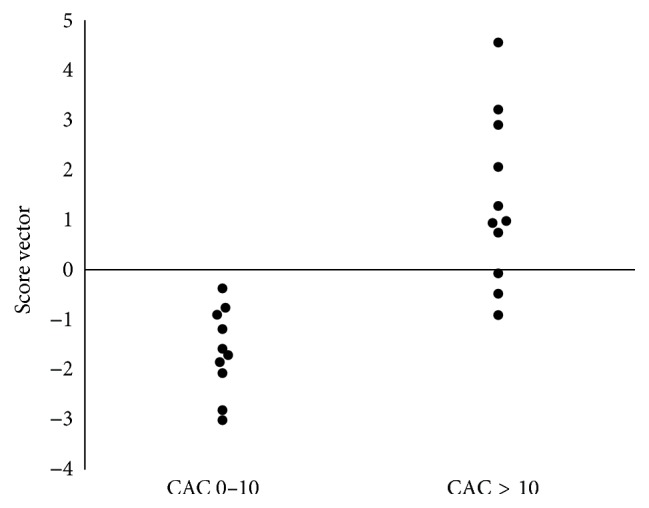
OPLS score vectors model 3. In the left column are the OPLS scores for patients with CAC 0–10 and in the right column the OPLS scores for patients with CAC > 10. In this model only one latent variable (vector) was identified. CAC: coronary artery calcification.

**Figure 7:**
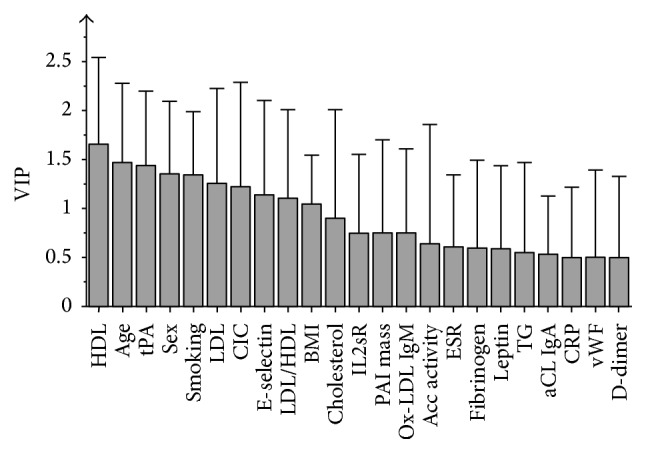
OPLS variable importance in projection (VIP) model 3. The numerical value for a variable represents an estimation of its importance in the model, that is, to what grade it discriminates between the two groups of patients. Whiskers illustrate the upper limit of bootstrapped confidence intervals. Acc activity: accumulated disease activity. aCL: anti-cardiolipin. BMI: body mass index. CIC: circulating immune complexes. CRP: c-reactive peptide. ESR: erythrocyte sedimentation rate. HAQ: health assessment questionnaire. HDL: high-density lipoproteins. IL2sR: soluble receptor of interleukin-2. IMT: carotid intima media thickness. LDL: low-density lipoproteins. ox-LDL: oxidized LDL. PAI: plasminogen activator inhibitor-1 mass. TG: triglycerides. tPA: tissue plasminogen activator. vWF: von Willebrand factor.

OPLS model 3 is presented in Figures 6 and 7. Similar to model 2, it included only baseline variables, but IMT and plaque were omitted. *R* ^2^ was 0.58, sensitivity 67%, and specificity 80%. As for model 2, all variables except HDL and leptin were positively related to high CAC.

## 4. Discussion

In this long-term follow-up of patients with seropositive RA for over 30 years, we found CAC in two of three patients. As expected, the most important baseline variables implicating a high level of CAC 13 years later were ultrasound signs of early atherosclerosis and the traditional cardiovascular risk factors as higher age, male sex, blood lipids, and smoking. Less expected was that levels of inflammation at baseline and accumulated inflammation before baseline were informative in OPLS analysis discriminating patients with high future CAC from patients with low future CAC. The importance of inflammation is further expressed in the OPLS model including not only baseline variables, but also variables reflecting current inflammatory status at follow-up and change in inflammatory variables during the 13-year period of follow-up. That model separated patients with high CAC from patients with low CAC at follow-up considerably precise, with a sensitivity of 89% and a specificity of 85%. Somewhat unexpected, exclusion of the baseline ultrasound variables from this model reduced the accuracy in separation only slightly. The loading plot for model 1 (Figure 3) visualizes how this is possible. DAS28 is in the loading plot located close to IMT and plaque, illustrating that these variables of high importance have a similar variation and to a great extent provide the same information to the OPLS model. It is also worth noting that when information on baseline IMT and plaque was excluded from this model, it was still more accurate than the baseline model including ultrasound variables (model 2). This implicates that variables reflecting inflammatory status over time actually are more useful in separating the two groups of patients than baseline IMT and plaque are. Furthermore, delta values reflecting change of inflammatory activity during follow-up (i.e., ESR, haptoglobin) surpassed the importance of baseline inflammation.

In our study, univariate correlations were found between CAC and the inflammatory variables swollen joint count, DAS 28, and ESR at follow-up. None of the previous studies have reported an association between CAC and clinical activity assessed by joint count. CAC has in previous studies been associated with duration [14, 15] and severity of disease [16], but not with measures of current joint inflammation. The results in earlier studies regarding association between markers of inflammation and CAC have been divergent, as for other measures of atherosclerosis [19]. Neither did a study of the incidence and progression of CAC in patients with RA find these measures associated with inflammatory activity or disease characteristics [27].

In previous studies, prevalence of CAC was associated with ESR in the studies where it was measured [14, 18, 28]. Several other laboratory markers of inflammation have also been studied. Rho et al. [18] and Giles et al. [16] reported that CAC was associated with IL-6, a proinflammatory cytokine that stimulates the inflammatory cascade. We analysed IL-6 at baseline but found no association with CAC, possibly due to the low-sensitive method of analysis. Also CRP was analysed with low-sensitive method and not shown to be associated with CAC. However, neither did the previous studies in patients with RA report an association between CRP and CAC [14–18, 28]. The lack of association between CAC and CRP in contrast to ESR and IL-6 indicates that the association between atherosclerosis, calcification, and inflammation is complex and that individual cytokines might represent different pathogenic pathways.

One previous study has assessed carotid arteries by ultrasound and CAC [29] in patients with RA. In that study, several patients with significantly increased IMT and plaques, indicating an elevated risk for cardiovascular event, had no CAC. Although this finding could have a temporal explanation with plaques and increased IMT preceding calcification, it could be another indication of early atherosclerosis and calcification being regulated in different manners. Nevertheless, the accumulated grade of long-term inflammation was related to CAC in the present study and to IMT in previous studies [6, 30, 31]. High inflammatory activity was also associated with more unstable coronary plaques [32] and carotid plaques [33] in patients with RA, associations analogous to the fact that patients with a high grade of inflammation have an increased risk of cardiovascular events [34]. This leads to the clinical implication that lowering the burden of inflammation is beneficial not only regarding the manifestations of RA, but also regarding atherosclerosis and risk of cardiovascular disease.

High levels of LDL and low levels of HDL are associated with high CAC in the general population [35, 36]. Our findings are adherent to this fact, with low HDL at baseline as one of the most important factors associated with high CAC at follow-up. Also levels of LDL have a relation to future high CAC, although not of the same magnitude as HDL. Triglycerides were of less importance in the present results, in contrast with previous studies by Chung et al., where triglycerides but not HDL and LDL were associated with CAC [14, 27]. One possible explanation to differences between studies measuring blood lipids in patients with RA is the relation between inflammation and lipid levels. The levels of total cholesterol, HDL, and LDL are known to decrease with increasing inflammation. This means that patients being at high risk of atherosclerosis and cardiovascular events due to high grade of inflammation have paradoxically low levels of lipoproteins [37]. Statin treatment is also an obvious confounder when measuring blood lipids, but no patient was on statin treatment at baseline in our study.

According to the OPLS models, treatment with statins and antihypertensive drugs at follow-up was associated with a high CAC. This association is probably confounded by indication. Treatment with these drugs is started preferably in patients with high cardiovascular risk or after a cardiovascular event, making these medications more common in patients susceptible of having high CAC. In a meta-analysis of studies on the general population, no effect of statin treatment on the progression of CAC was demonstrated [38]. In contrast, atorvastatin decreased the progression of CAC in a study of patients with SLE [39]. This is another indication that different pathways of inflammation might affect the process of arterial calcification in different manners.

The study has several strengths. The first is the prospective design and long period of follow-up. The study population is another strength of this study. The study patients were seropositive, less than 66 years old at baseline, and diagnosed at the same clinic in the same period of time but were otherwise unselected. Although this makes the group of patients heterogenous in terms of disease activity, medication, complications, and comorbidity, it makes the study population representative of Caucasian patients with seropositive, long-term RA and homogenous in terms of disease duration. No patients in the follow-up had diabetes or chronic kidney disease, two well-known causes of arterial calcification.

Some limitations of the study must be pointed out. First, the number of patients in this follow-up is very small. It is likely that patients with a moderate grade of calcification differ from patients with high or very high grade, but this could not be analysed due to the small number of patients eligible for investigation in this follow-up study. The distribution of patients lost to follow-up was probably not random. It is plausible that the missing patients were from the group with high CAC rather than low; five of the deceased patients had suffered from cardiovascular events in the period of follow-up. The patients who refused to participate due to severe disability are also probably at risk of having high CAC, since a relation between CAC and disease activity or severity has been shown in both the present study and previous studies [14, 16]. The number of men was small at baseline and more than half of them were lost during the period of follow-up. Only four men participated in this study, making the results more indicative of the female population.

## 5. Conclusion

In patients with long-term RA, the vast majority had coronary artery calcification (CAC). High CAC (Agatston score > 10) was significantly associated with a higher grade of current inflammation. OPLS discriminant analysis of variables assessed at baseline 13 years earlier discriminated between patients with high CAC and patients with low CAC with a sensitivity of 73% and specificity of 82%. However, inclusion of measures of inflammatory activity at follow-up and the change between baseline and follow-up considerably increased the discrimination between patients with high and low CAC (sensitivity 89% and specificity 85%). Apparently, the burden of inflammation is important for the understanding of atherosclerosis in RA.

## Figures and Tables

**Table 1:** Characteristics of patients with RA at baseline and at follow-up after 13 years.

	Baseline	Follow-up
	*n* = 39	*n* = 22
*Demographic*		
Age (range)	53 (38–65)	63 (51–78)
Female, number (%)	30 (77)	18 (81.8)

*Cardiovascular risk factors*		
Diabetes, number (%)	1 (2.6)	0
Current smoking, number (%)	8 (20.5)	0
Ever smoking, number (%)	17 (43.6)	7 (31.8)
Systolic blood pressure mmHg	na	140 (128–149)
Diastolic blood pressure mmHg	na	80 (78–90)
Antihypertensive treatment, number (%)	8 (20.5)	10 (45.5)
Cholesterol mmol/L	5.2 (4.6–6.3)	5.7 (4.7–6.5)
LDL mmol/L	3.2 (2.8–4.2)	3.5 (2.5–4.4)
HDL mmol/L	1.4 (1.1–1.7)	1.5 (1.3–2.0)
Triglycerides mmol/L	1.1 (0.8–1.8)	1.1 (0.93–1.5)
Statin treatment, number (%)	0	3 (13.6)
BMI kg/m^2^	22.7 (21.6–25.8)	na
BMI < 20 kg/m^2^, number (%)	5 (12.8)	na
BMI > 30 kg/m^2^, number (%)	3 (7.7)	na

*Inflammatory variables*		
ESR mm/h	22 (10–32)	15 (9–27)
CRP mg/L	10 (5–19)	5 (5–17)
Haptoglobin mg/L	1.6 (1.0–2.1)	1.6 (1.0–1.9)
Tender joint count	na	2 (0–3)
Swollen joint count	na	0 (0–3)
DAS28	na	3.3 (2.3–3.9)
HAQ	na	0.63 (0.25–1.5)
Global VAS mm	na	28 (12–41)
Positive RF	39 (100)	na

All numbers are presented as median (Q1–Q3), except when indicated else.

LDL: low-density lipoproteins. HDL: high-density lipoproteins. BMI: body mass index. ESR: erythrocyte sedimentation rate. CRP: C-reactive peptide. DAS28: 28-joint disease activity score. HAQ: health assessment questionnaire. VAS: visual analogue scale. RF: rheumatoid factor. na: not analysed.

**Table 2:** Inflammatory variables at follow-up.

Variable	CAC 0–10	CAC > 10	*p* value
ESR (mm/h)	9.0 (6–14)	26 (14–30)	<0.01
CRP (mg/L)	5.0 (5.0–9.5)	8.0 (5.0–20)	ns
DAS28	2.31 (2.05–3.30)	3.61 (3.29–4.41)	<0.01
Swollen joints	0 (0-1)	2 (0–3)	<0.05
Tender joints	0 (0–5)	2 (2-3)	ns
HAQ	0.5 (0.2–1.5)	0.9 (0.3–1.7)	ns

Values are presented as median (Q1–Q3). ns = not significant (*p* > 0.05).

CAC: coronary artery calcification score. ESR: erythrocyte sedimentation rate. CRP: C-reactive peptide. DAS28: 28-joint disease activity score. HAQ: health assessment questionnaire.
